# Prediction of Pest Insect Appearance Using Sensors and Machine Learning

**DOI:** 10.3390/s21144846

**Published:** 2021-07-16

**Authors:** Dušan Marković, Dejan Vujičić, Snežana Tanasković, Borislav Đorđević, Siniša Ranđić, Zoran Stamenković

**Affiliations:** 1 Faculty of Agronomy in Čačak, University of Kragujevac, Cara Dušana 34, 32102 Čačak, Serbia; stanasko@kg.ac.rs; 2 Faculty of Technical Sciences Čačak, University of Kragujevac, Svetog Save 65, 32102 Čačak, Serbia; dejan.vujicic@ftn.kg.ac.rs (D.V.); sinisa.randjic@ftn.kg.ac.rs (S.R.); 3 Institute Mihailo Pupin d.o.o., Volgina 15, 11060 Belgrade, Serbia; bora@impcomputers.com; 4 IHP-Leibniz-Institut für Innovative Mikroelektronik, Im Technologiepark 25, 15236 Frankfurt Oder, Germany; stamenko@ihp-microelectronics.com

**Keywords:** machine learning, pest insect appearance, temperature and relative humidity sensors, precision agriculture

## Abstract

The appearance of pest insects can lead to a loss in yield if farmers do not respond in a timely manner to suppress their spread. Occurrences and numbers of insects can be monitored through insect traps, which include their permanent touring and checking of their condition. Another more efficient way is to set up sensor devices with a camera at the traps that will photograph the traps and forward the images to the Internet, where the pest insect’s appearance will be predicted by image analysis. Weather conditions, temperature and relative humidity are the parameters that affect the appearance of some pests, such as *Helicoverpa armigera*. This paper presents a model of machine learning that can predict the appearance of insects during a season on a daily basis, taking into account the air temperature and relative humidity. Several machine learning algorithms for classification were applied and their accuracy for the prediction of insect occurrence was presented (up to 76.5%). Since the data used for testing were given in chronological order according to the days when the measurement was performed, the existing model was expanded to take into account the periods of three and five days. The extended method showed better accuracy of prediction and a lower percentage of false detections. In the case of a period of five days, the accuracy of the affected detections was 86.3%, while the percentage of false detections was 11%. The proposed model of machine learning can help farmers to detect the occurrence of pests and save the time and resources needed to check the fields.

## 1. Introduction

Determining and monitoring the values of factors that affect agricultural production is of great importance for achieving the best possible quality and yield. These factors may be related to sowing, harvesting, or the period in between when it is necessary to undertake appropriate agro-technical measures. Such processes related to factor optimization in agriculture belong to the research and development area known as precision agriculture.

The growth of information technologies has enabled the establishment of a more precise farm management system. Precision agriculture represents the strategic application of information technologies to gather data from multiple sources with the aim of making decisions related to agricultural production, marketing, finance and personnel. Precision agriculture aims to achieve increased crop quality, improved sustainability, environmental protection and rural development through new skills [1].

The expansion of wireless sensor networks (WSN) has greatly contributed to the development of precision agriculture. The addition of appropriate sensors and software could provide crops with exactly what they need, which leads to productivity optimization. Sensors from a given location can obtain real-time data about the conditions of soil, crops and weather. Information extracted from images of the area obtained by a satellite or an airplane is also very helpful for decision-making [2].

With the further development of hardware and communication technologies, the Internet of Things (IoT) technology has emerged as a continuation of WSN. IoT is also applied in precision agriculture [3]. IoT enables the collection of sensor data and their transmission to the Internet, where it is processed using machine learning and can provide adequate information relevant to crop management [4].

The motivation comes from the need to predict pest insect appearance in a timely manner at many different localities in a whole region, based on available data that are collected on temperature and relative humidity. The goal was not to determine the insect population density but to predict (using meteorological parameters) when the first insects would occur in order to provide users sufficient time to react and reduce the pest insect population.

The sections in this paper are organized as follows. The second section presents the state-of-the-art in the field. The third section describes the methodology applied to detect and predict the development of pest insects in agricultural production. The fourth section describes the hardware platform used for data collection and processing. A description of the results and a discussion are presented in the fifth section. Finally, the paper ends with concluding remarks.

## 2. State-of-the-Art

Applying the concepts of precision agriculture and the remote detection of pest insects, farmers can undertake appropriate measures to suppress the appearance of insects or reduce the insect population. Insect populations above the economic threshold can cause substantial damage to plants and thus reduce yields. On the other hand, information on the occurrence and number of pests is important to reduce the use of pesticides, decrease inputs and protect the environment. The number of pests at an observed locality was commonly checked by visual observation of sticky surfaces in insect traps and counting the caught insects. Such an initial setup would require human engagement for the almost daily or weekly checking of trap sticky bases, which is a highly time-consuming job and requires some resources such as fuel and vehicles, because traps can be installed/located over a wide geographical area. To overcome this problem, the technology on which precise agriculture relies is beginning to be used for monitoring the situation inside the set insect trap wherever it is located, from a remote position. Accordingly, article [5] shows an overview of techniques as well as sensors for the automatic detection and monitoring of pest insects. The main emphasis was on pest identification using infrared sensors, audio sensors and image-based classification. Recent achievements regarding machine-learning techniques were also presented.

The processing of large amounts of data obtained from remote sensing devices in precision agriculture is increasingly oriented towards application of the machine learning (ML) approach. One of the examples of the use of ML in agriculture is presented in [6], where predictions of crop yield and predictions of nitrogen status, as the main mineral nutrients required for plant growth and development, are presented.

There are many examples of predicting crop yields based on input parameters where the basis is weather data. Article [7] presents different models of machine learning that enabled the analysis of data obtained on soil, climatic conditions and water regime and, as a result, crop yields were predicted and the applied machine learning approaches were compared. In article [8], the input data of soil and crop properties were used to predict yield. ML algorithms can be used to predict alfalfa yield [9], maize yield and nitrate loss [10], and to assess the seasonal nitrogen status in maize [11,12], carrot yield mapping [13], soil suitability for growing individual crops [14] and peach tree nutrients at the local level [15].

A particularly significant application of ML models in the field of agronomy is found in the derivation of new values based on the images of fields, plants and pests as input data. Reference [16] uses ML models to classify crops and monitor plant growth status. Using the ML approach, it is also possible to classify plant leaves based on image analysis [17], plant segmentation [18] or palm tree classification, depending on whether they are infected with plant diseases [19].

The estimation of new values based on available weather data using the ML model is shown in [20], where soil temperature was determined. In Reference [21], the wheat yield was also estimated using, in addition to climate data, satellite images provided in a time series.

Reference [22] presents the use of data from various sources to make certain predictions using the ML model to improve the management of a smart farm. In [23], deep learning techniques were applied in the field of agronomy.

Machine learning can have significant applications in the identification of insects in crops based on images from the site. Knowing the type and number of insects is of great importance to protect crops and preserve yields. In [24], a description of research on the topic of automatic insect detection using ML techniques is given. The paper [25] presents the potential of ML for crop protection with the help of early detection of plant diseases or harmful insects. An ML weed identification system is shown in [26] to increase the plant protection potential.

Sticky traps can be used to detect and monitor the number of insects in a certain locality, and in the paper [27] insects were recognized based on images of the current state of the traps using convolutional neural networks.

Reference [28] presents a prediction model for *Helicoverpa armigera*, which represents the number of insects weekly. The model is based on weather data, while its validation was performed using data collected on insects caught in pheromone traps. Temperature, relative humidity, and the number of hours of sunshine were singled out as weather factors that influence the appearance of *Helicoverpa armigera*.

According to [29], the occurrence of *Helicoverpa armigera* insects was introduced by a model based on satellite data of surface temperature, where the ML technique provided an estimation of insect population dynamics after the first detection of their larvae. In [30], an approach for the prediction of *Helicoverpa armigera* in future periods is presented according to the expected climate changes.

Existing examples usually represent a total number of insects under certain conditions observed for a longer time. The aim of this paper is the presentation of an ML model, which can predict (with the help of meteorological data) the appearance of insects in a certain region on daily basis. This paper presents an ML model that uses the relative humidity in addition to temperature as input data to predict pest insect appearance and to optimize the utilization of resources.

## 3. Methodology

We propose a methodology for the prediction of pest insect appearance, which uses the environmental parameters (temperature and relative humidity) as input data and machine learning for data processing and output generation. According to [28], we initially determined our input parameters, the day of the year, temperature and relative humidity; values that we can obtain in our region. The idea was (based on existing data and without additional investments) to help users (owners of small plantations in different locations) to predict insect appearance and allow them sufficient time to control the dynamics of insect growth. Data on *Helicoverpa armigera* insects caught in traps with light lamps were collected from 17 locations in the northern part of Serbia, Vojvodina province, during 2019 and 2020. In parallel, daily data on the number of trapped insects and data from meteorological stations located in their immediate vicinity were collected [31]. From the monitored environmental data, the temperature and relative humidity were recorded on a daily basis during the season in successive series representing the input dataset.

The three to five days observation period was selected according to pest development. At the beginning of vegetation, adults (butterflies and moths) emerge for an extended period of approximately three weeks. Oviposition begins two to six days later with the expulsion of a single egg during the night. Female fecundity (the total number of eggs) is around 3000. This species is highly polyphagous. Larval development is harmful (detrimental) for cultivated plants. Larval feeding results in fruits boring, rotting and plant decay. Therefore, it is extremely important to register the appearance of moths in the field and suppress the occurrence of the insects by applying chemicals at two stages (moth and egg). Larvae are usually hidden within stem or fruits and are protected by plant tissue, which makes the spraying impossible. The suppression of moths and eggs will lower the loss and damage in the field. This is the most important step of *Helicoverpa armigera* population control and crop protection. Our approach predicts the appearance of insects in a period of three to five days when the user can react and prevent the growth of the insect population. It is a customized method guided by real requirements, which estimates the accuracy of the proposed ML model for one, three, and five days in a row.

We consider the confusion matrix of our ML model and try predicting the insect appearance (confirmed value ‘1’), similar to True Positive (TP) values on the given day, in the next three, or the next five, days. If the pest insects appear in these periods, a prediction hit is observed.

Examples of ML models were generated using the Scikit-learn package. Scikit-learn is an open-source Python library that provides support for the implementation of machine learning algorithms. It is based on NumPy and SciPy, which are Python libraries for scientific computation. Due to the wide application of Python, Scikit-learn has gained more popularity. Scikit-learn is characterized by comprehensive coverage of ML models. The implementation of ML model algorithms is optimized for efficient execution on computing resources. Scikit-learn also has great community support for documentation, bug tracking and quality assurance. Within Scikit-learn, the presentation of input and output data is uniform. There is also a fixed procedure for the fitting model so that it is possible to change methods without excessive effort [32]. 

The ML algorithms used to form multiple model variants were K-Nearest Neighbors, Support Vector Machines with kernel = ‘rbf’ (RBF SVM), Support Vector Machines with kernel = ‘poly’ (Poly SVM), Decision Tree, Random Forest, Multi-layer Perceptron classifier (Neural Net), Ada Boost, Gaussian Naive Bayes (G Naive Bayes), and Quadratic Discriminant Analysis (QDA) [33]. Table 1 shows the input variables and optimal parameter values for individual ML models.

Based on the data collected from the analyzed locations, an overall dataset was formed, which was used in the first step for the validation and comparison of ML algorithms. The input variables, in addition to the day of the year, represent the values for temperature (T) and relative humidity (RH) in the last ten days in a row. As shown in Table 1, twenty input values are denoted by T_k_ and RH_k_, respectively, where the number k ranges from day d to d-9 in the past (from k = d, k = d-1… to k = d-9). Prior to the learning process, the complete dataset was randomly divided into two parts: the first part of 75% for training and the second part of 25% for validation. Since in the second case we would like to keep the data form in a time series, we separated the two sets in the same proportions but so that we kept the array of data in the same order as they appeared successively during the season by days. 

The accuracy score and confusion matrix are used as outputs to verify the results. The accuracy score is a function that calculates the accuracy of correct predictions [33] and can be represented as:

(1) $$Accuracy=\frac{1}{n}\sum_{i=1}^{n-1}1(P_{i}=T_{i}),$$

where P_i_ is the predicted value, T_i_ is the true value, *n* is the number of samples and 1(x) is the indicator function.

The confusion matrix is one of the metrics used for determining the accuracy of classification when training ML models. In the case of binary problems, the number of true negatives, false positives, false negatives and true positives is obtained. Therefore, Table 2 presents a confusion matrix of the size 2 × 2, that is, the case of classification into two groups [34] is shown, where there are in fact two values, true (1) and false (0).

The accuracy of prediction can also be calculated using the confusion matrix as:

(2) $$Accuracy=\frac{TN+TP}{TN+FP+FN+TP}.$$

In addition to reaching the accuracy, the confusion matrix was used to represent the validation of the results in the ML model, enabling the observation of how many positive values were actually affected (TP) and how many positive values were incorrectly detected (FP). This way of analyzing the results was convenient because the new correct and false values can be calculated over an extended range that includes three and five days. The obtained values represent the new parameters in Equation (2) for calculating the new accuracy.

In addition to accuracy, we also considered the F1 score since real data represents an imbalanced dataset. The F1 score can be calculated according to Equation (3):

(3) $$F1\;score=2\times \frac{Precision\times Recall}{Precision+Recall},$$

where Precision would be obtained by Equation (4)

(4) $$Precision=\frac{TP}{TP+FP}$$

and Recall would be obtained by Equation (5).

(5) $$Recall=\frac{TP}{TP+FN}.$$

We compared the presented ML models calculating the accuracy defined by Equation (2), which includes four elements of the confusion matrix (binary classification) and also we took into consideration F1 scores defined by Equation (3).

## 4. Hardware Platform

The collection of temperature and relative humidity values was achieved using the Pessl Instruments Hygroclip sensor, that is, via the PT1000 1/3 Class B temperature sensor and the ROTRONIC Hygromer IN-1 humidity sensor. The accuracy with the standard setting was ±0.8% RH/±0.1 °C, while the accuracy with high precision was ±0.5% RH/0.1 °C. The measuring range was from 0% to 100% RH and from 100 to 200 °C. The output signal is intended for the serial port RS485 [35].

The presented sensor is a part of the iMeteos system, where iMeteos1 and iMeteos 3.3 meteorological stations were used at certain localities. The iMeteos 3.3 system, as well as other products from the iMeteos group, are intended for monitoring data with the help of various sensors by providing the possibility of measuring, logging and sending data to platforms on the Internet. The basic unit of iMeteos 3.3 contains a box consisting of electronic components, a battery, and with an attached solar panel and a dual antenna. The basis of the system is the iMeteos 3.3 board, which contains a 32 bit ARM Cortex M3 processor and a Real Time Operating System (RTOS). One of its main features is operational reliability as it has a flash memory of 8 MB and can store data for up to approximately a month. A 6 V and 4 Ah battery is connected directly to the iMeteos 3.3 board, as well as a solar panel to the appropriate connectors. 

In addition to the role of the data logger, the system contains a UMTS/CDMA modem and can send data to the FieldClimate platform. There is also a SIM card holder so that data transfer to the platform on the Internet can be achieved via cellular base stations using appropriate protocols. The iMeteos 3.3 board has 12 direct inputs and up to 600 sensors can be connected. Certain types of sensors need to be connected to special dedicated connectors, such as a wind speed sensor, leaf humidity sensor, or hygroclip sensors that measure temperature and relative humidity [36,37].

## 5. Validation of ML Model

**Figure 1:**
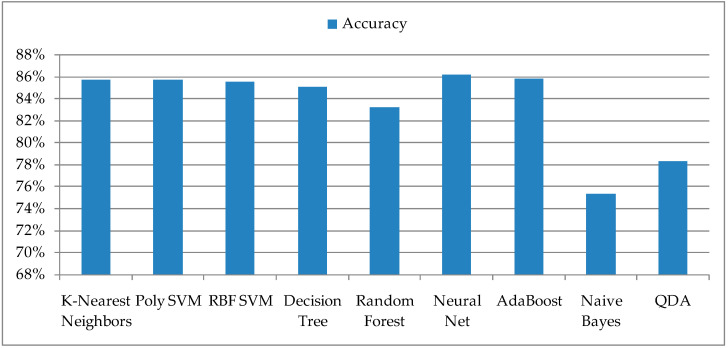
Accuracy of the used ML algorithms.

The proposed ML models for classification were compared after the training and validation of the dataset that included the date, temperature, and relative humidity. The output of the model has only two possible values, 1 and 0, whether there was a detection or no detection of insects on an observed day. The entire dataset was first loaded and randomly distributed to the training dataset and the dataset for its validation. The accuracy of individual ML models is presented in Figure 1. It can be observed that the last two ML models, Naive Bayes and QDA, have the lowest accuracy and therefore can be excluded from further analyses.

**Figure 2:**
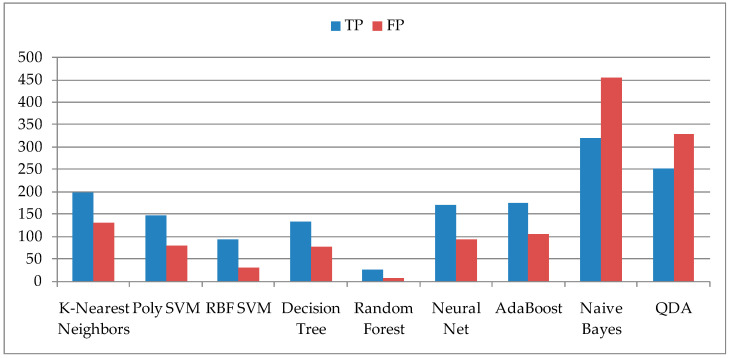
Values for True Positive (TP) and False Positive (FP) after model validation.

The number of days when insects are detected in the season is smaller than the number of other days when there are no insects, so days with no insects have a significant influence due to the higher prevalence on determining accuracy. It is much more important for users to get information about the days when the prediction was performed, so it was necessary to extract the results from the confusion matrix of the validated models. Based on the confusion matrix, the results are shown in Figure 2, which represent the TP (True Positive) when the prediction is confirmed and the FP (False Positive) when the false or incorrect prediction is confirmed. The number of FPs in the two ML models, Naive Bayes and QDA, which had the lowest accuracy, is higher than the number of TPs, which also excludes them from the set of ML models. The ML model Random Forest, as well as the RBF SVM, have low TP values, so they were not suitable for the prediction of pest insect appearance. 

After validation of the ML model, Nearest Neighbors, Poly SVM, Decision Tree, Neural Net and AdaBoost were selected from the original set. To test the ML model that would evaluate the occurrence of insects, a dataset was used, which consisted of several seasons characterized by a smaller number of TPs, that is, a smaller number of days when the occurrence was detected compared to the previous validation dataset. The test dataset represented temperature and relative humidity by days, in time series, during the season gathered from different locations. The testing dataset was not randomly extracted from the whole dataset but represents the real situation at these locations, and there is a somewhat smaller number of days when insects appeared compared to the dataset on which the validation was performed. Applying the proposed ML model over the testing dataset, we have calculated the accuracy and elements of the confusion matrix. The FP has higher values than in the validation process (Table 3).

Precisely because we took the testing dataset in the time series, it is possible to adjust the presented models so that we can use them to predict the day when the insects will appear. Prediction of *Helicoverpa armigera* (D_HA_) appearance in one day was counted if there was a real occurrence of an HA insect on that day (R_HA_ = 1) and if there was a prediction for insect occurrence (P_HA_ = 1), where the total number of D_HA_, marked as numD_HA_, is equal to TP. The daily prediction accuracy of AD_HA_ can be calculated according to:

AD_HA_ = numD_HA_/numR_HA,_ (6)

where AD_HA_ is the prediction accuracy on a daily basis, numD_HA_ is the number of days when the prediction of the insect occurrence was confirmed and numR_HA_ is the number of days when the insects appeared.

We can extend our model by observing three days in one variant or even five days in another solution. First, using the selected ML models, we predicted the output data for the selected test dataset, the metrics of which are shown in Table 1. After that, we checked the predicted results using an extended test range of three and five days. To validate our model, we exported the predicted values for every day into a series and compared them with the real values of that day, and of the next three and five days. 

The appearance of the HA insect can be considered confirmed if the predictive value was indicated on that day and a real insect would appear on that day or in the next two days. The pseudocode that shows the mentioned way of observing the results within three days, which means enough time for user intervention, is:

IF P_d_ = 1 and (R_d_ = 1 or R_d+1_ = 1 or R_d+2_ = 1)

THEN D_HA_3d_ = 1 

ENDIF.

According to the previous pseudocode, P_d_ represents the predictive value of the insect HA on day d. R_d_ is the real occurrence for day d, R_d+1_ is the real occurrence for the next day and R_d+2_ is for the next two days. D_HA_3d_ represents the prediction of the pest insect appearance in the observed period of three days and has a positive value if there was a prediction of the insect’s occurrence for that day and the actual occurrence of insects in that or the next two days. The same principle was used for a period of five days and is shown in the following pseudocode: 

IF P_d_ = 1 and (R_d_ = 1 or R_d+1_ = 1 or R_d+2_ = 1 or R_d+3_ = 1 or R_d+4_ = 1)

THEN D_HA_5d_ = 1 

ENDIF.

The accuracy of the prediction of the pest insect appearance in a period of 3 days AD_HA_3d_ is calculated similarly according to:

AD_HA_3d_ = numD_HA_3d_/numR_HA,_ (7)

where numD_HA_3d_ is the number of days when the prediction of the appearance of HA insects was confirmed within the observed period of three days and numR_HA_ is the number of days when the insects appeared.

**Figure 3:**
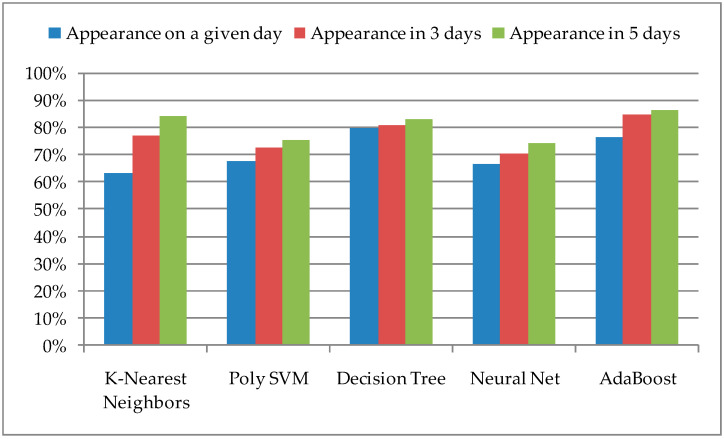
Prediction of the pest insect appearance according to an extended method with selected ML algorithms.

The obtained values for the successful prediction of the pest insect’s appearance on the same day, a period of three days, and a period of five days are presented in Figure 3. 

A model based on the Decision Tree algorithm has shown the best results in the case of successful detection on the same day, that is, the greatest value of TP, which is 79.8%. After increasing the observation range, the accuracy of successful prediction also increases, with the fact that for the mentioned Decision Tree algorithm it is a slightly higher value, but for a period of five days, the new value of the accuracy is 83.1%. The largest increase in accuracy after expanding the range is with models with the K-Nearest Neighbors algorithm, where the new value is 84.2%. However, the biggest increase in the extended method was obtained with AdaBoost, where for a period of three days the accuracy was 84.7%, and for a period of five days 86.3%.

**Figure 4:**
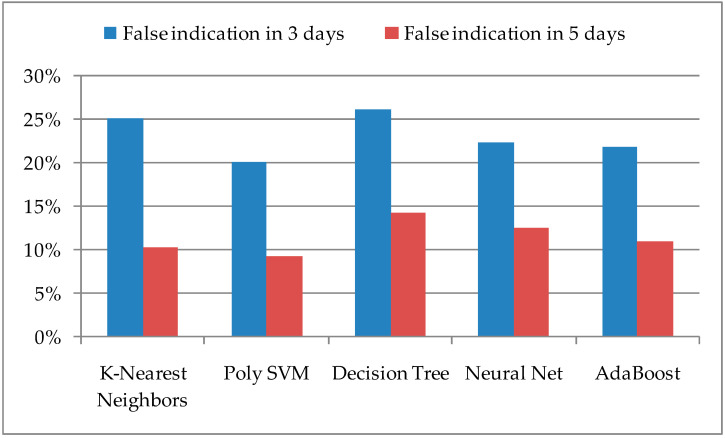
False prediction of the pest insect appearance according to the observed period and ML algorithms.

In addition to the accuracy of successful predictions, it is important to consider the number of false predictions. Their value indicates the expectation of occurrences in a certain locality, which in fact will not happen, and requires unnecessary user engagement. Therefore, it is necessary to reduce the value of FP to a lower value within the ML model, which can be achieved in some way if the same periods of three and five days are observed, especially if false prediction occurs successively for several days in a row. The percentage values of false predictions with the number of real insect occurrences are shown in Figure 4. It can be observed that Poly SVM and AdaBoost have the lowest values for the proposed ML algorithms.

**Figure 5:**
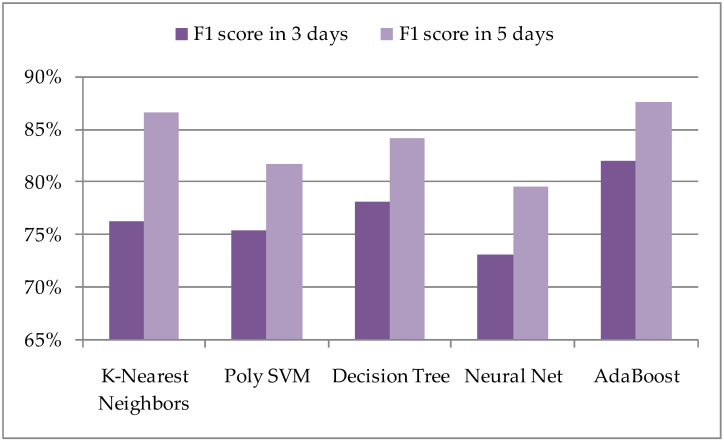
F1-scores according to an extended method with selected ML algorithms.

Since the number of days when insects appear is relatively smaller compared to the number of days when they do not appear, which represents real data, such a dataset can be considered unbalanced. Thus, in addition to accuracy, the F1 scores of selected ML models with an extended method could also be considered (Figure 5).

For the given application, and using the extended method, the K-Nearest Neighbors and AdaBoost algorithms generate the highest scores. The ML model with AdaBoost algorithm stands out, which, in addition to the highest accuracy of the extended ML model, also has one of the smallest values of false detections; for AdaBoost, it is about 11% in the observed period of five days.

## 6. Discussion of Results

In addition to visiting the locations, one of the ways to detect insects is the use of sensor-based equipment and advanced information and communication technologies. Usually, such a system involves the use of a camera that would capture insects’ images and whereby images from a remote location, with the support of WSN or IoT, would be transferred to the Cloud platform where they would be analyzed. By applying ML algorithms to the collected data, that is, to the images, certain insects can be recognized [27]. In the case when the equipment and required resources that would enable direct recognition of insects are not available, other solutions could be considered. The ML model could be used to perform predictions based on the values of temperature and relative humidity. These are the values of weather data available at all meteorological stations and represent the parameters that influence the appearance of pest insects such as *Helicoverpa armigera*.

In an interesting research paper [28], the number of HA at the weekly level was predicted and it was shown that the important parameters of weather conditions that affect the occurrence of HA are the number of sunshine hours, temperature, and relative humidity. The mean values of small moths caught in sticky traps were recorded and compared with output values predicted by the model on a weekly basis. The proposed model showed satisfactory validation and that, in addition to temperature, a significant parameter that affects the occurrence of HA is relative humidity. 

In several previous studies [38,39,40], machine learning has been used for the prediction of the insect population density or the number of trapped insects. The aim of this study was to predict the appearance on the site of the insect species *Helicoverpa armigera* as an economically highly significant pest. The advantage of the proposed model is in the early detection of the presence of *H. armiger*, which provides the farmer with the opportunity to monitor the insect population over the next three to five days, use an insecticide, and reduce the population growth.

Since weather parameters can be collected from several localities, it is also important to consider their application in the prediction of the pest insect’s appearance on a daily basis, in addition to the possible prediction of their number for the observed period on a weekly basis. It would be important for users to get an indication at which sites and on which days the HA insects could potentially occur. In that way, special attention would be paid to those places and timely reactions could be taken by users to prevent the spread of pest insects.

The presented results were obtained based on models with ML algorithms, where the prediction of the pest insect’s appearance for a certain day was performed based on the values of temperature and relative humidity taken for the previous ten days in the array. Testing of the model is performed with data that make up the days in the series during one season, where it can happen that, in such a set, the number of insect occurrences varies and is not so large compared to the total number of days in the season. Based on the testing of the ML model, it could be noticed that, in such cases, the results are obtained with satisfactory accuracy, but also with a larger number of false predictions of the pest insect’s appearance. To make this case more acceptable, we presented the data in a time series and introduced an extended method that observes several days in a row. In this way, the model interprets the condition as true if there is a prediction for the insect occurrence and the insects really appear in the next three days.

The input data to our ML model represent the temperature and relative humidity over the last ten days. Future work may consider the model’s behavior when varying importance for different days, as well as the application of ML models on sequences such as HMM, semi-Markov CRF, RNN, and LSTM to preserve temporal dependence.

## 7. Conclusions

In this article, we presented a machine learning based prediction model that indicates the possibility of pest appearance using the temperature and relative humidity as environmental parameters. It reduces the number of terrain visits and saves human and other resources. On the other hand, the additional equipment to monitor volatile insect traps is not necessary.

The advantages of the presented ML model for the prediction of the insect appearance come from the fact that it uses easily accessible parameters (temperature and relative humidity) as input values. In addition, the accuracy of the model increases as a period of three or five days is observed when the predicted value can be expected, which gives users sufficient time to organize their activities and reduce the insect population. The presented model lacks flexibility, since input specifications depend on the observed insect. In other words, it is necessary to take into account different conditions and specific data recognition patterns for selected insects. The proposed ML model can indicate the potential situation in the field and provide farmers an optimal platform for work. In addition, the farmers can better plan their activities in a certain period (a few days or weeks). Then they will have the opportunity to assess their priorities, to determine which sites to visit first, as well as to postpone the application of insecticides in case precipitation is expected.

## Figures and Tables

**Table 1:** Input variables and parameters in used ML models.

Input Variables	Name	Parameters
21 variables (DayInYear, T_d_, T_d-1_, T_d-2_, T_d-3_, T_d-4_, T_d-5_, T_d-6_, T_d-7_, T_d-8_, T_d-9_, RH_d_, RH_d-1_, RH_d-2_, RH_d-3_, RH_d-4_, RH_d-5_, RH_d-6_, RH_d-7_, RH_d-8_, RH_d-9_)	K-Nearest Neighbors	n_neighbors = 3, weights = ‘uniform’, algorithm = ‘auto’, leaf_size = 30, metric = ‘minkowski’, p = 2
	Poly SVM	C = 10, degree = 3, gamma = ‘scale’, break_ties = False, cache_size = 200, decision_function_shape = ‘ovr’, kernel = ‘poly’
	RBF SVM	C = 1, degree = 3, gamma = 2, break_ties = False, cache_size = 200, decision_function_shape = ‘ovr’, kernel= rbf
	Decision Tree	criterion = ‘gini’, splitter = ‘best’, max_depth = 5, min_samples_split = 2, min_samples_leaf = 1, class_weight = None
	Random Forest	n_estimators = 10, criterion = ‘gini’, max_depth = 5, min_samples_split = 2, min_samples_leaf = 1, class_weight = None
	Neural Net	hidden_layer_sizes = (100,), activation = ‘relu’, solver = ‘adam’, alpha = 1, batch_size = ‘auto’, learning_rate = ‘constant’, power_t = 0.5, learning_rate_init = 0.001, max_iter = 1000, shuffle = True, early_stopping = False
	AdaBoost	algorithm = ‘SAMME.R’, base_estimator = None, learning_rate = 1.0, n_estimators = 50, random_state = None
	G Naive Bayes	priors = None, var_smoothing = 1 × 10^−9^
	QDA	priors = None, reg_param = 0.0, store_covariance = False, tol = 0.0001

**Table 2:** The confusion matrix for binary classification.

	Predicted Negative	Predicted Positive
Actual Negative	TN	FP
Actual Positive	FN	TP

where: TN (True Negative) is the number of correct negative predictions, FP (False Positive) is the number of incorrect positive predictions, FN (False Negative) is the number of incorrect negative predictions, and TP (True Positive) is the number of correct positive predictions.

**Table 3:** Accuracy and elements of the confusion matrix after checking the ML model on the testing dataset.

ML Model	Accuracy	TN	FP	FN	TP
K-Nearest Neighbors	84.2%	1536	243	67	116
Poly SVM	87.3%	1589	190	59	124
Decision Tree	86.6%	1553	226	37	146
Neural Net	87.1%	1587	192	61	122
AdaBoost	87.1%	1569	210	43	140

## Data Availability

Not applicable.
